# Supplementary material for: Gut Microbiota Profile and Changes in Body Weight in Elderly Subjects with Overweight/Obesity and Metabolic Syndrome
Source: Microorganisms. 2021 Feb 10;9(2):346. doi: 10.3390/microorganisms9020346 (PMC7916506; doi:10.3390/microorganisms9020346)
Supplement: Supplementary file 1 [file microorganisms-09-00346-s001.pdf]

microorganisms

MDPI

Supplementary Materials: Gut Microbiota Profile and Changes in Body Weight in Elderly Subjects with Overweight/Obesity and Metabolic Syndrome

Alessandro Atzeni, Serena Galié, Jananee Muralidharan, Nancy Babio, Francisco José Tinahones, Jesús Vioque, Dolores Corella, Olga Castañer, Josep Vidal, Isabel Moreno-Indias, Laura Torres-Collado, Rebeca Fernández-Carrión, Montserrat Fitó, Romina Olbeyra, Miguel Angel Martínez-González, Monica Bulló* and Jordi Salas-Salvadó *

Table S1. Differences in richness metrics, Chao1, Shannon and Simpson, between tertiles of baseline body mass index.

	**Differences in Chao1**	
	Tertile 1	Tertile 2
Tertile 2	0.95 [#]	-
Tertile 3	0.95	0.95
	**Differences in Shannon**	
	Tertile 1	Tertile 2
Tertile 2	0.82	-
Tertile 3	0.82	0.82
	**Differences in Simpson**	
	Tertile 1	Tertile 2
Tertile 2	0.92	-
Tertile 3	0.92	0.92

[#] False discovery rate *P*-value tested by Wilcoxon rank sum test.

Table S2. Differences in beta diversity distances, Bray-Curtis, Jaccard, Weighted Unifrac, Unweighted Unifrac, between tertiles of baseline body mass index.

			**Differences in Bray-Curtis distance**			
	Df	Sums of Sqs	Mean Sqs	F.Model	R2	Pr(>F)
Tertile	2	0.762	0.381	1.062	0.006	0.318
Residuals	361	129.461	0.359		0.994	
Total	363	130.223			1.000	
			**Differences in Jaccard distance**			
	Df	Sums of Sqs	Mean Sqs	F.Model	R2	Pr(>F)
Tertile	2	0.863	0.431	1.033	0.006	0.323
Residuals	361	150.728	0.417		0.994	
Total	363	152.591			1.000	
			**Differences in Weighted Unifrac distance**			
	Df	Sums of Sqs	Mean Sqs	F.Model	R2	Pr(>F)
Tertile	2	0.004	0.002	0.633	0.003	0.535
Residuals	361	1.267	0.003		0.996	
Total	363	1.271			1.000	
			**Differences in Unweighted Unifrac distance**			
	Df	Sums of Sqs	Mean Sqs	F.Model	R2	Pr(>F)
Tertile	2	0.218	0.109	0.904	0.005	0.716
Residuals	361	43.552	0.121		0.995	
Total	363	43.770			1.000	

Df, degrees of freedom; Sqs, squares; Pr(>F), significance on F-test. Differences in beta diversity distances were tested using PERMANOVA test. Permutations: free; number of permutations 999; terms added sequentially (first to last).

Table S3. Variability in beta diversity distances among tertiles of baseline body mass index.

**Bray-Curtis distance**						
	Df	Sum Sq	Mean Sq	F	N.Perm	Pr(>F)
Groups	2	0.0002	0.0001	0.050	999	0.955
Residuals	361	0.911	0.002			
**Jaccard distance**						
	Df	Sum Sq	Mean Sq	F	N.Perm	Pr(>F)
Groups	2	0.00008	0.00004	0.044	999	0.953
Residuals	361	0.316	0.0009			
**Weighted Unifrac distance**						
	Df	Sum Sq	Mean Sq	F	N.Perm	Pr(>F)
Groups	2	0.0009	0.0004	0.439	999	0.644
Residuals	361	0.357	0.001			
**Unweighted Unifrac distance**						
	Df	Sum Sq	Mean Sq	F	N.Perm	Pr(>F)
Groups	2	0.0005	0.0002	0.066	999	0.939
Residuals	361	1.323	0.004			

Df, degrees of freedom; Sq, square; N.Perm, number of permutations; Pr(>F), significance on F-test. Permutation test for homogeneity of multivariate dispersions performed to statistically test variability among groups. Permutation: free; number of permutations: 999; response: distances.

Table S4. Pairwise comparisons of baseline body mass index tertiles mean dispersion.

**Bray-Curtis distance**			
	Tertile 1	Tertile 2	Tertile 3
Tertile 1	-	0.885 [#]	0.871
Tertile 2	0.879	-	0.743
Tertile 3	0.871	0.747	-
**Jaccard distance**			
	Tertile 1	Tertile 2	Tertile 3
Tertile 1	-	0.823	0.929
Tertile 2	0.838	-	0.769
Tertile 3	0.931	0.768	-
**Weighted Unifrac distance**			
	Tertile 1	Tertile 2	Tertile 3
Tertile 1	-	0.374	0.910
Tertile 2	0.392	-	0.435
Tertile 3	0.927	0.438	-
**Unweighted Unifrac distance**			
	Tertile 1	Tertile 2	Tertile 3
Tertile 1	-	0.762	0.738
Tertile 2	0.779	-	0.977
Tertile 3	0.735	0.972	-

[#] Observed P-value below diagonal. permuted *P*-value above diagonal, calculated using t-test performed on the pairwise group dispersions.

Table S5. Results of log normalized *Firmicutes*-to-*Bacteroidetes* ratio and *Prevotella*-to-*Bacteroides* ratio between tertiles of baseline body mass index.

	N	Mean	Std dev	Min	Max
Log2_FB_ratio					
Tertile 1	121	0.861	1.388	−5.500	3.536
Tertile 2	122	0.864	1.298	−4.917	2.964
Tertile 3	121	0.915	1.310	−3.917	2.839
Log2_PB_ratio					
Tertile 1	121	−0.224	2.786	−11.459	10.071
Tertile 2	122	−0.146	2.543	−9.515	6.104
Tertile 3	121	−0.758	3.154	−12.788	5.687

Log2_FB_ratio one-way ANOVA (F (2, 361) = 0.064, *P* = 0.938).
Log2_PB_ratio one-way ANOVA (F (2, 361) = 1.388, *P* = 0.332).

Table S6. Results of log normalized *Firmicutes*-to-*Bacteroidetes* ratio and *Prevotella*-to-*Bacteroides* ratio between tertiles of changes in body weight after 12-month intervention.

	N	Mean	Std dev	Min	Max
Log2_FB_ratio					
Tertile 1	115	0.783	1.367	−2.96	5.22
Tertile 2	115	1.046	1.223	−2.74	4.21
Tertile 3	115	0.759	1.358	−3.54	4.11
Log2_PB_ratio					
Tertile 1	115	−0.289	2.756	−12.47	6.00
Tertile 2	115	−0.270	2.576	−12.70	6.06
Tertile 3	115	−0.295	2.778	−11.46	5.69
Delta_FB_ratio					
Tertile 1	115	0.041	1.404	−4.05	4.59
Tertile 2	115	−0.229	1.325	−7.16	2.44
Tertile 3	115	0.116	1.478	−6.86	3.83
Delta_PB_ratio					
Tertile 1	115	−0.164	3.297	−12.62	12.47
Tertile 2	115	0.034	2.838	−13.26	9.72
Tertile 3	115	0.293	3.399	−11.79	11.46

Log2_FB_ratio one-way ANOVA (F (2, 342) = 1.680, *P* = 0.188)
Log2_PB_ratio one-way ANOVA (F (2, 342) = 0.003, *P* = 0.997).
Delta_FB_ratio one-way ANOVA (F (2, 342) = 1.931, *P* = 0.147).
Delta_PB_ratio one-way ANOVA (F (2, 342) = 0.598, *P* = 0.550).

Table S7. Differential abundant ASVs, and correspondent assigned genera, identified by DESeq2 in gut microbiota, between tertiles of baseline body mass index.

ASV ID	baseMean	log2FoldChange	lfcSE	stat	pvalue	padj	Genus
			T1 vs T2				
5b1b613f2b8a8224ffb8f4df91585f76	10.099	29.200	3.568	8.185	2.7×10^{-16}	1.2×10^{-12}	Prevotella 2
			T1 vs T3				
6c03f9ce7c4e3251103817d98a644563	0.442	21.623	3.585	6.032	1.6×10^{-9}	6.9×10^{-6}	Bacteroides
			T2 vs T3				
6c03f9ce7c4e3251103817d98a644563	0.442	29.927	3.540	8.453	2.8×10^{-17}	1.2×10^{-13}	Bacteroides
5b1b613f2b8a8224ffb8f4df91585f76	10.099	−18.876	3.541	−5.33	9.8×10^{-18}	2.1×10^{-14}	Prevotella 2

lfcSE, log fold change Standard Error; stat, Wald test statistic; padj, Benjamini-Hochberg adjusted *P*-value.

Table 8. Differential abundant ASVs, and correspondent assigned genera, identified by DESeq2 in gut microbiota, between tertiles of changes in body weight after 12-month intervention.

ASV ID	baseMean	log2FoldChange	lfcSE	stat	*p*-value	padj	Genus
			T1 vs T2				
de204b44fc231cd402652f4d87380471	0.234	23.705	3.671	6.457	1.1×10^{-10}	7.3×10^{-7}	Prevotella 9
d7cc2fea278ab0156b4e6e72dcdbe327	0.103	22.482	3.671	6.123	9.2×10^{-10}	2.0×10^{-6}	uncultured
c2a9c6e2349c0166d4d1f321354baae3	0.095	22.355	3.671	6.088	1.1×10^{-9}	2.0×10^{-6}	Lachnospiraceae UCG-001
733ac0a6c00a457793ea9c9d1a428aa9	0.102	22.323	3.671	6.080	1.2×10^{-9}	2.0×10^{-6}	NA
cc5d6923fc7f1124160533a11bcfbb76	0.078	22.072	3.671	6.011	1.8×10^{-9}	2.5×10^{-6}	Bacteroides
5b1b613f2b8a8224ffb8f4df91585f76	8.046	−18.829	3.670	−5.13	2.9×10^{-7}	3.3×10^{-4}	Prevotella 2
			T1 vs T3				
de204b44fc231cd402652f4d87380471	0.234	24.512	4.303	5.697	1.2×10^{-8}	8.3×10^{-5}	Prevotella 9
d7cc2fea278ab0156b4e6e72dcdbe327	0.103	23.473	4.303	5.454	4.9×10^{-8}	1.2×10^{-4}	uncultured
c2a9c6e2349c0166d4d1f321354baae3	0.095	23.330	4.303	5.421	5.9×10^{-8}	1.2×10^{-4}	Lachnospiraceae UCG-001
cc5d6923fc7f1124160533a11bcfbb76	0.078	23.103	4.303	5.368	7.9×10^{-8}	1.2×10^{-4}	Bacteroides
733ac0a6c00a457793ea9c9d1a428aa9	0.102	23.018	4.303	5.349	8.8×10^{-8}	1.2×10^{-4}	NA
d00c1161004d40e7b94bbbda74a867cf	0.355	22.966	4.303	5.105	3.3×10^{-7}	3.7×10^{-4}	Bacteroides
			T2 vs T3				

d00c1161004d40e7b94b bbda74a867cf	0.356	28.676	3.830	7.488	7.0×10^{-14}	1.2×10^{-10}	Bacteroides
5b1b613f2b8a8224ffb8f 4df91585f76	8.046	26.343	3.831	6.876	6.1×10^{-12}	4.7×10^{-9}	Prevotella 2
c24bf620a7e511901684 4066114196fc	0.070	−23.898	3.832	−6.237	4.5×10^{-10}	1.7×10^{-7}	Sutterella
68ec1aefa0103ae766daf c814b38894b	0.142	−24.646	3.832	−6.432	1.3×10^{-10}	5.0×10^{-8}	Bacteroides
26cb357d46b7cb7c7fc6 0dec29530cba	0.077	−24.767	3.832	−6.464	1.0×10^{-10}	4.4×10^{-8}	Prevotella 2
89595c082e6cf4a47edb b41a7d13bd3a	0.095	−24.929	3.832	−6.506	7.7×10^{-11}	3.5×10^{-8}	Dialister
a10c31f88f53ba0b7319 de39d21e0ee6	0.099	−24.931	3.832	−6.506	7.7×10^{-11}	3.5×10^{-8}	Prevotella 9
21ab743a71ced7896203 e4bfeca1289f	0.113	−24.988	3.832	−6.521	6.7×10^{-11}	3.5×10^{-8}	Prevotella 9
579a75cbf324508b5a74 d6533dd9169c	0.181	−25.015	3.832	−6.528	6.6×10^{-11}	3.5×10^{-8}	Bacteroides
fb5d414fd6bd7d6dc753 f0b889a3c473	0.167	−26.198	3.832	−6.837	8.1×10^{-12}	5.0×10^{-9}	Bacteroides
88bff18bedc7d38cd6ff3 b46201c90d0	0.167	−26.198	3.832	−6.837	8.1×10^{-12}	5.0×10^{-9}	Bacteroides
a188c60c7eab567a12ae 4318c1a54694	0.107	−26.339	3.832	−6.874	6.2×10^{-12}	4.7×10^{-9}	Bacteroides
9e7912a029eca5c8e694 a06acaa15ef3	0.111	−28.364	3.832	−7.4	1.3×10^{-13}	1.3×10^{-10}	Bacteroides
a58697475d24d4d265ea a922b346d6f5	0.088	−28.490	3.832	−7.435	1.0×10^{-13}	1.2×10^{-10}	Prevotella 2
4c6f7e347c7157738bafc b4ac98c385f	0.103	−28.547	3.832	−7.450	9.3×10^{-14}	1.2×10^{-10}	NA
eb1e06232c80ba2efd64 dfa172203d5c	0.171	−28.813	3.832	−7.520	5.5×10^{-14}	1.2×10^{-10}	Prevotella 9
d49ea76b83aacea8576b fc30b28f0caa	0.165	−29.180	3.832	−7.615	2.6×10^{-14}	9.0×10^{-11}	Bacteroides
02500c8e7dff746267b9 b6542b9f5140	0.225	−33.036	3.832	−8.622	6.6×10^{-18}	4.5×10^{-14}	Bacteroides

lfcSE, log fold change Standard Error; stat, Wald test statistic; padj, Benjamini-Hochberg adjusted *P*-value.

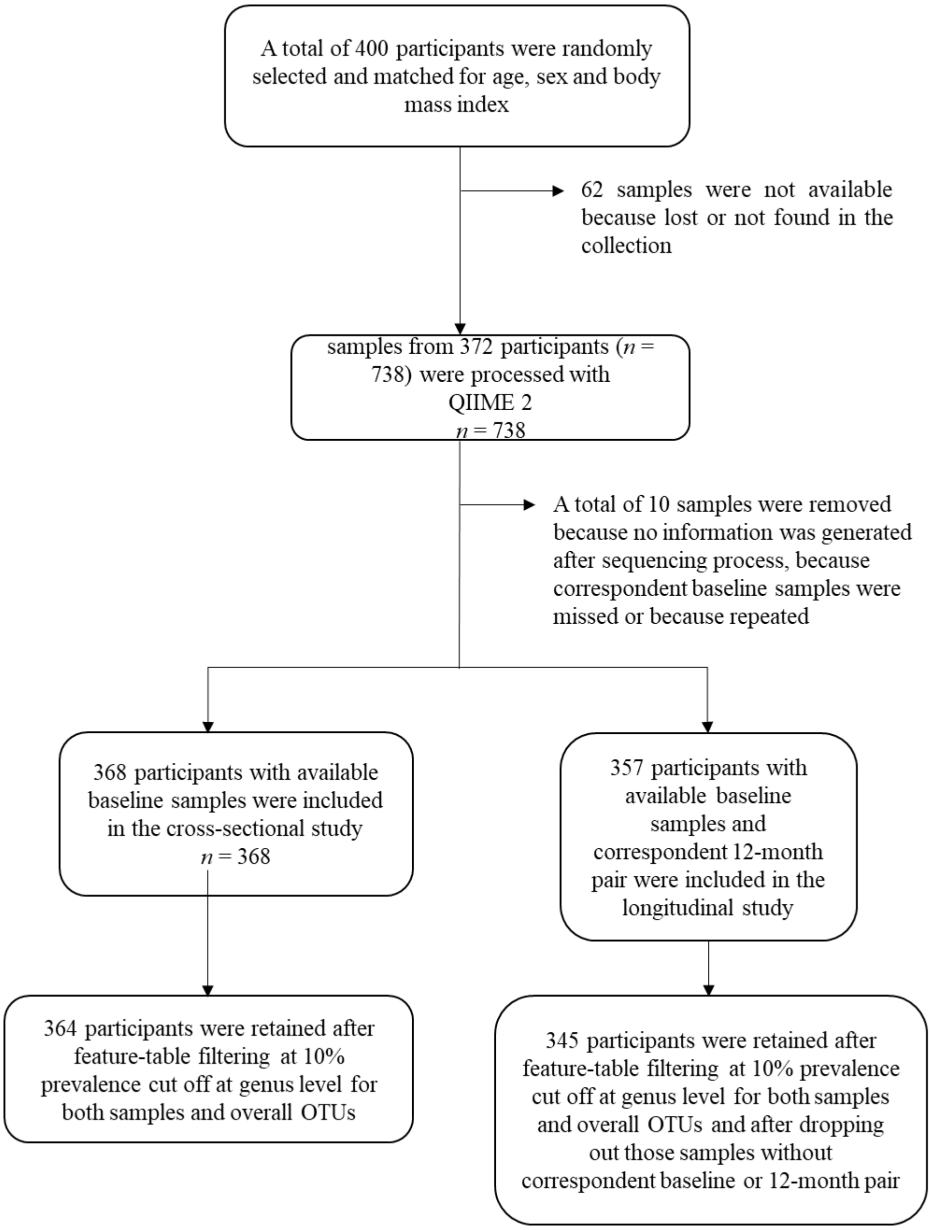

Figure S1. Flowchart of study participants.
